# Cost of illness of chronic kidney disease in Lebanon: from the societal and third-party payer perspectives

**DOI:** 10.1186/s12913-022-07936-0

**Published:** 2022-05-01

**Authors:** Mabel Aoun, Elie Helou, Ghassan Sleilaty, Rony M. Zeenny, Dania Chelala

**Affiliations:** 1 Department of Nephrology, Faculty of Medicine, Saint-Joseph University, Beirut, Lebanon; 2 Department of Nephrology, Saint-George Hospital, Ajaltoun, Lebanon; 3 Department of Urology, Faculty of Medicine, Saint-Joseph University, Beirut, Lebanon; 4 Unit of biostatistics, Faculty of Medicine, Saint-Joseph University, Beirut, Lebanon; 5 Pharmacy Director, American University of Beirut Medical Center, Beirut, Lebanon; 6 Department of Nephrology at Hotel-Dieu de France Hospital, Faculty of Medicine, Saint-Joseph University, Beirut, Lebanon

**Keywords:** Cost-of-illness, Societal perspective, Chronic kidney disease, Kidney transplantation, Hemodialysis, Peritoneal dialysis, Pharmacoeconomics, Health costs, Burden of illness

## Abstract

**Background:**

Chronic kidney disease (CKD) is the 12^th^ leading cause of death worldwide. Cost-of-illness studies of CKD are scarce in developing countries. This study aims to estimate the cost of illness of all stages of CKD in Lebanon, from early stages until dialysis and kidney transplantation. The secondary objective is to identify factors related to the highest financial burden.

**Methods:**

This is a cross-sectional study of CKD patients who presented to two nephrology clinics during November 2020. Their medical and administrative records were reviewed for collection of demographics, CKD characteristics, direct medical costs (medications, diagnostic tests, hospitalizations, inpatient care, outpatient care), direct non-medical costs (transportation) and indirect costs (productivity losses) for one year. Kruskal Wallis test was used to compare the costs between different CKD stages and categories. Logistic regression analysis was used to evaluate risk factors associated with costs.

**Results:**

The sample included 102 non-dialysis CKD patients, 40 hemodialysis, 8 peritoneal dialysis and 10 transplant patients. Their mean age was 66.74 ± 15.36 years, 57.5% were males and 42.5% diabetics. The total median cost per year of CKD across all categories was assessed to be 7,217,500 Lebanese Pounds (3,750,000–35,535,250; 1 $USD = 1515 LBP in 2019) from the societal perspective and 5,685,500 LBP (2,281,750- 32,386,500) from the third-party payer perspective. Statistical analysis showed a higher total cost in hemodialysis (*p* < 0.001), higher cost of medications in transplant (*p* < 0.001) and higher cost in technique modality in peritoneal dialysis (*p* < 0.001). In a sub-analysis of hemodialysis patients, dialysis vintage negatively correlated with total societal cost (*r* = -0.391, *p* = 0.013); the regression analysis found diabetes as a risk factor for higher cost (OR = 2.3; 95%CI: 0.638,8.538; *p* = 0.201). In the subcategory of CKD-ND patients, age correlated with total societal cost (r = 0.323, *p* = 0.001); diabetes and coronary artery disease were significantly associated with higher total cost (OR = 2.4; 95%CI: 1.083,5.396; *p* = 0.031; OR = 3.7; 95%CI: 1.535,8.938; *p* = 0.004).

**Conclusions:**

This cost of illness study showed a high burden of hemodialysis and peritoneal dialysis cost compared to transplant and non-dialysis CKD patients. It revealed a significantly higher cost of medications in transplant patients. Health policies should target interventions that prevent end-stage kidney disease and encourage kidney transplantation.

**Supplementary Information:**

The online version contains supplementary material available at 10.1186/s12913-022-07936-0.

## Background

Chronic Kidney Disease (CKD) contributes to more than one million deaths yearly worldwide and is considered to be the 12^th^ leading cause of global death [1]. CKD and its associated cardiovascular mortality and morbidity are a huge burden on the individual and society. Moreover, the economic impact of CKD is reported to be high, both in developed and developing countries [2]. A recent study from the United States showed that CKD costs increase with the decline of the estimated Glomerular Filtration Rate (eGFR) and the presence of diabetes [3]. In Germany, they identified dialysis treatment and hospitalizations as the major cost drivers in CKD patients [4]. In Italy, an analysis of different types of costs revealed that the direct non-medical and indirect costs were as important as the direct medical costs in CKD [5]. In Mexico, a national study pointed out to the large burden of CKD and called for new strategies to prevent the cost of all kidney disease stages [6]. The CARRS study, conducted in South-Asian countries, found out that cardiometabolic diseases including CKD are associated with a high economic burden, leading to financing distress in non-insured patients [7]. In the Middle East, CKD was also emphasized as a high burden on health systems and authors called for renal registries to better assess the economic burden of this disease [8].

In Lebanon, economic studies about the cost of illness of chronic kidney disease are scarce. A paper from 2016 tried to analyze the societal cost of illness of hemodialysis patients before the era of upgrade of water treatment quality and the increase of the bundled fee [9–11]. On another note, evaluation of costs in CKD non-dialysis (CKD ND) patients is lacking. The CKD prevalence in Lebanon was estimated at 12.5% recently [12]. This is higher than the 9.1% estimated globally in 2017 [13]. The dialysis prevalence in Lebanon is also among the highest worldwide estimated at 777 patients per million population (pmp) against 410 dialysis patients pmp worldwide [11, 13]. The country is also currently embarking on the biggest inflation of its history and lacks a national database that could help measuring the national cost of CKD. Therefore, there is a need to estimate this cost from a sample of the population and identify what stages of CKD and associated comorbidities can impact directly the health expenditure of the population.

Cost of illness studies use various methodologies and perspectives depending on the context and the purpose of the study [14]. In Lebanon, healthcare services are covered by the Ministry of Public Health (MOPH), the national social security fund (NSSF), military instances, the private insurances and/or out-of-pocket (OOP). There is a fixed bundle fee for dialysis covered by third-party payers, reimbursed to hospitals. This fixed fee does not take into consideration the indirect costs. Therefore, it would be relevant to evaluate the costs of CKD in Lebanon from both the societal and the third-party payer perspectives. A cost-of-illness study is crucial to evaluate the most important components of CKD costs, whether direct medical, direct non-medical or indirect costs. Identifying factors that are related to the highest economic burden of kidney illness can help decision-makers set their priorities when faced with low resources and deliver appropriate health policies [2, 4, 5, 7, 15–18].

This study aims to estimate the cost of illness of all stages of CKD in Lebanon, from the early stages until dialysis and kidney transplant. The secondary objective is to identify factors related to the highest financial burden.

## Methods

### Study setting, participants

This is a cross-sectional study of all CKD patients visiting two nephrologists affiliated with Saint-Joseph University and working in four distinct Lebanese in-hospital clinics. Patients were included within two weeks during November 2020. Data were collected retrospectively from the medical and administrative records of patients. Patients of any CKD stage were included if they were above 18 years old and if their records included comprehensive data about their medications, diagnostic tests, third-payers, hospitalizations, work loss and other opportunity costs as well as transportation. Transplant patients and peritoneal dialysis (PD) patients coming to the clinics were included. We included as well all hemodialysis (HD) patients followed by one of the nephrologists. Knowing that there is a fixed bundled fee for all hemodialysis patients in the country, any dialysis unit's evaluation would be a good estimate of other hemodialysis patients' costs. Patients who lacked any of the necessary information in their files and who were followed simultaneously by other physicians or were admitted to facilities where data were unreachable were excluded.

### Types of costs and different perspectives of cost-of-illness evaluation

Different types of costs were measured from the societal and third-party payer perspectives (Table 1). The opportunity cost, in simple words, is what the patient is missing while busy with his disease. The loss of productivity is an opportunity cost.

**Table 1:** Different types of costs from two perspectives

	Direct medical costs: medications, diagnostic tests (laboratory and imaging), inpatient and outpatient care	Direct non-medical costs: transportation costs, social services	Indirect costs: productivity losses or opportunity costs due to morbidity
Societal Perspective	All costs covered by the patient and third-party payers	All costs	All costs
Third-Party Payer Perspective	Covered costs	Not included	Not included

### Data collection

Variables were retrospectively collected from medical records for one year between June 1, 2019 and June 1, 2020. Data included age, sex, third-party payer (NSSF, MOPH, Insurance, OOP, Internal Security Forces, Army and/or Cooperative of Civil Servants), diabetes, hypertension, smoking, coronary artery disease (CAD), cause of CKD, serum creatinine and eGFR, urine albumin to creatinine ratio, dialysis vintage and vascular access (catheter or fistula) if on dialysis, duration of transplantation if transplanted, all medications and total cost of medications (including erythropoietin stimulating agents), all blood tests and costs, all imaging tests and costs, hospitalizations causes and costs, opportunity costs (productivity loss), transportation costs (specifically for hemodialysis patients), nursing costs (for hemodialysis patients). Costs of medications were retrieved from MOPH website public drugs price list. Costs of blood and imaging tests were calculated based on the different third-party payers' lists of laboratories and hospitals. Costs were reported in Lebanese Pounds (LBP), one United States (US) dollar is equivalent to 1515 LBP at the time of the study.

### Definitions

The glomerular filtration rate (eGFR) was estimated in all patients using the 2009 Chronic Kidney Disease Epidemiology collaboration (CKD-EPI) formula. CKD stages were defined based on the Kidney Disease: Improving Global Outcomes (KDIGO) 2012 Clinical Practice classification [19]: stage 1, eGFR > 90 ml/min with albuminuria > 30 mg/g; stage 2, eGFR 60–89 ml/min with albuminuria > 30 mg/g; stage 3a, eGFR 45–59 ml/min; stage 3b, eGFR 30–44 ml/min stage 4, eGFR 15–29 ml/min and stage 5 non-dialysis, eGFR < 15 ml/min.

### Sample size

The sample size was calculated using Epi Info 7 StatCalc functions for a population survey. Since the prevalence rate of CKD varies between 9 and 12% [12, 13], we estimated the minimal sample to be 160 people with a confidence level of 95% and a margin of error of 5%.

### Ethical considerations

The Ethics Committee of Hotel-Dieu de France and Saint-Joseph University approved the study proposal (CEHDF 1719). All data were collected anonymously and handled confidentially, aligning with the declaration of Helsinki.

### Statistical analysis

Statistical analyses were performed using the Statistical Package for Social Sciences (SPSS), version 23.0. Although this study had the main aim to be descriptive, further analysis was performed. Continuous data were reported as means and standard deviation (SD) if normally distributed and median and interquartile (IQR) if data were skewed. Categorical data were expressed as numbers and percentages. Kruskal–Wallis test was used to compare the costs of different groups. Spearman rho test was used to assess the correlation between total costs and continuous variables such as age and dialysis vintage. Logistic regression analysis was used to assess the association between costs (by dividing them into two groups based on the median) and different categorical variables and comorbidities. *P*-value < 0.05 indicates statistical significance.

## Results

### General characteristics

A total of 160 patients were included: 102 CKD-non dialysis, 40 hemodialysis, 10 transplant and 8 peritoneal dialysis patients. Their general characteristics are summarized in Table 2. Their mean age was 66.74 ± 15.36 years and 57.5% were males. The median number of years with a kidney transplant was 6.5 years (2.5, 14.75) and it ranged between one and 21 years. The median dialysis vintage of PD and HD patients were 36 years (29,49) and 49 years (26.5,96) respectively and they ranged between 13 and 50 months for PD and one and 21 years for HD. Six out of the 40 HD patients had a permanent catheter. Most of the CKD-ND patients had an eGFR < 60 mL/min and their median urine albumin to creatinine ratio was 397 mg/g (Table 3).

**Table 2:** General characteristics of different kidney disease categories

	**Total ** ***N* = 160**	**CKD-ND** ***N* = 102**	**PD** ***N* = 8**	**HD** ***N* = 40**	**Transplant** ***N* = 10**
**Age**					
Mean ± SD	66.74 ± 15.36	68.31 ± 14.43	63.38 ± 17.73	68.68 ± 13.43	45.70 ± 16.08
Median (IQR)	69 (60,78)	70 (62,79)	66.5 (59.3,73.3)	71.5 (61.3,79.5)	48 (29,58.5)
**Sex M/F, n(%)**	92/68 (57.5/42.5)	56/46 (54.9/45.1)	5/3 (62.5/37.5)	26/14 (65/35)	5/5 (50/50)
**eGFR**					
Mean ± SD	NA	37.23 ± 24.67	NA	NA	64.8 ± 23.08
Median (IQR)		33 (17,49.25)			64.5 (44.3,84.5)
**Cause of CKD, n(%) **					
Diabetic	68 (42.5)	48 (47.1)	2 (25)	16 (40)	2 (20)
Unknown etiology	23 (14.4)	12 (11.8)		9 (22.5)	2 (20)
Glomerulonephritis	24 (15)	12 (11.8)	4 (50)	5 (12.5)	3 (30)
PKD	9 (5.6)	4 (3.9)		5 (12.5)	
TIN	9 (5.6)	8 (7.9)		1 (2.5)	
NS/Vascular	9 (5.6)	7 (6.9)	2 (25)		
One kidney/FSGS	8 (5)	6 (5.9)		2 (5)	
Obstructive	2 (1.3)				2 (20)
Nephronophtisis	2 (1.3)			1 (2.5)	1 (10)
Others	6 (3.8)	5 (4.9)		1 (2.5)	
**CKD stages**					
Stage 1		7 (6.9)			
Stage 2	NA	8 (7.8)	NA	NA	NA
Stage 3a		13 (12.7)			
Stage 3b		29 (28.4)			
Stage 4		28 (27.5)			
Stage 5 ND		17 (16.7)			
**Diabetes, n(%)**	79 (49.4)	58 (56.9)	2 (25)	16 (40)	3 (30)
**Hypertension, n(%)**	148 (92.5)	95 (93.1)	7 (87.5)	37 (92.5)	9 (90)
**CAD, n(%)**	50 (31.3)	35 (34.3)	2 (25)	12 (30)	1 (10)
**Smoking, n(%)**	54 (33.8)	44 (43.1)	3 (37.5)	6 (15)	1 (10)
**Number of medications per day**					
Mean ± SD	8.75 ± 3.38	8.47 ± 3.74	10.38 ± 2.83	9.3 ± 2.31	8.1 ± 3.2
Median (IQR)	9 (7,11)	8 (6,11)	9 (8.25,12.5)	10 (8,10)	7.5 (5.75,10.25)
**Frequency of blood tests per year**					
Mean ± SD	6.86 ± 6.4	3.49 ± 1.72	4 ± 1.19	16.5 ± 5.36	4.9 ± 3.48
Median (IQR)	4 (3,12)	3 (2,4)	4.5 (3,5)	15 (13,18)	3.5 (3,6)
**Number of hospitalizations**					
Mean ± SD	0.79 ± 1.43	0.5 ± 0.9	1 ± 2.07	1.65 ± 2.08	0.1 ± 0.31
Median (IQR)	0 (0,1)	0 (0,1)	0 (0,1)	1 (0,3)	0 (0,0)
**Third-Party Payer, n(%)**					
NSSF	79 (49.4)	48 (47.1)	3 (37.5)	21 (52.5)	7 (70)
MOPH	13 (8.1)	1 (1)			
CSC	12 (7.5)	9 (8.8)		3 (7.5)	
ISF	2 (1.3)	2 (2)			
Insurance	25 (15.6)	25 (24.5)			
Army	3 (1.9)	3 (2.9)			
NSSF + Insurance	14 (8.8)	9 (8.8)	3 (37.5)	5 (12.5)	
MOPH + Insurance	8 (5)	1 (1)		2 (5)	2 (20)
Out-of-pocket	3 (1.9)	3 (2.9)			
OOP + MOPH	1 (0.6)	1 (1)	2 (25)	9 (22.5)	1 (10)

*Note. NA* Not applicable, Third-party payers are multiple and can overlap: *NSSF* The national social security fund, *MOPH* The ministry of public health, *CSC* The Cooperative of Civil Servants, *ISF* The Internal Security Forces; Insurance means private insurances, *OOP* is out-of-pocket

Causes of kidney diseases: *PKD* Polycystic kidney disease, *TIN* Tubulointerstitial nephritis, *NS* Nephrosclerosis, *FSGS* Focal segmental glomerulosclerosis

**Table 3:** Characteristics of CKD-ND patients across different stages

	**CKD stages 1–2 ** **N = 15**	**CKD stages 3a-3b** ***N* = 42**	**CKD stages 4–5** **N = 45**
**Age**			
Mean ± SD	49.27 ± 16.19	70.14 ± 10.43	72.96 ± 12.02
Median (IQR)	48 (34,64)	71.5 (62.75,78.25)	72 (68,83.5)
**Sex M/F, n(%)**	6/9 (40/60)	23/19 (54.8/45.2)	27/18 (60/40)
**eGFR**			
Mean ± SD	83.4 ± 16.83	41.86 ± 8.38	17.51 ± 9.82
Median (IQR)	78 (70,100)	40.5 (34,49)	16 (10.5, 24)
**Urine albumin to creatinine ratio**			
Median (IQR)	260 (82.5, 1130)	318.5 (53.5, 600)	630 (64, 2000)
**Cause of CKD, n (%)**			
Diabetic Nephropathy	6 (40)	19 (45.2)	23 (51.1)
Unknown etiology	1 (6.7)	5 (11.9)	6 (13.3)
Glomerulonephritis	4 (26.7)	2 (4.8)	6 (13.3)
PKD	1 (6.7)	2 (4.8)	1 (2.2)
TIN	0	2 (4.8)	4 (8.9)
NS/Vascular	0	6 (14.3)	1 (2.2)
Others	3 (20.1)	6 (14.3)	4 (8.9)
**Diabetes, n(%)**	7 (46.7)	25 (59.5)	26 (57.8)
**Hypertension, n(%)**	12 (80)	41 (97.6)	42 (93.3)
**CAD, n(%)**	3 (20)	15 (35.7)	17 (37.8)
**Smoking, n(%)**	5 (33.3)	18 (42.9)	21 (46.7)
**Number of medications per day**			
Mean ± SD	4.5 ± 3.5	8.3 ± 2.9	9.9 ± 3.6
**Number of hospitalizations**			
Mean ± SD	0.3 ± 0.6	0.4 ± 0.8	0.6 ± 1
**Third-Party Payer, n (%)**			
NSSF	9 (60)	20 (47.6)	19 (42.2)
MOPH	0	0	1 (2.2)
CSC	2 (13.3)	1 (2.4)	6 (13.3)
ISF	0	0	2 (4.4)
Insurance	2 (13.3)	13 (31)	10 (22.2)
Army	0	3 (7.1)	0
NSSF + Insurance	2 (13.3)	3 (7.1)	5 (11.1)
MOPH + Insurance	0	1 (2.4)	0
Out-of-pocket	1 (6.7)	1 (2.4)	2 (4.4)

*Note*. *NA* Not applicable, Third-party payers are multiple and can overlap: *NSSF* The national social security fund, *MOPH* The ministry of public health, *CSC* The Cooperative of Civil Servants, *ISF* The Internal Security Forces; Insurance means private insurances, *OOP* is out-of-pocket

Causes of kidney diseases: *PKD* Polycystic kidney disease, *TIN* Tubulointerstitial nephritis, *NS* Nephrosclerosis, *FSGS* Focal segmental glomerulosclerosis

### Costs from the societal perspective

**Fig. 1:**
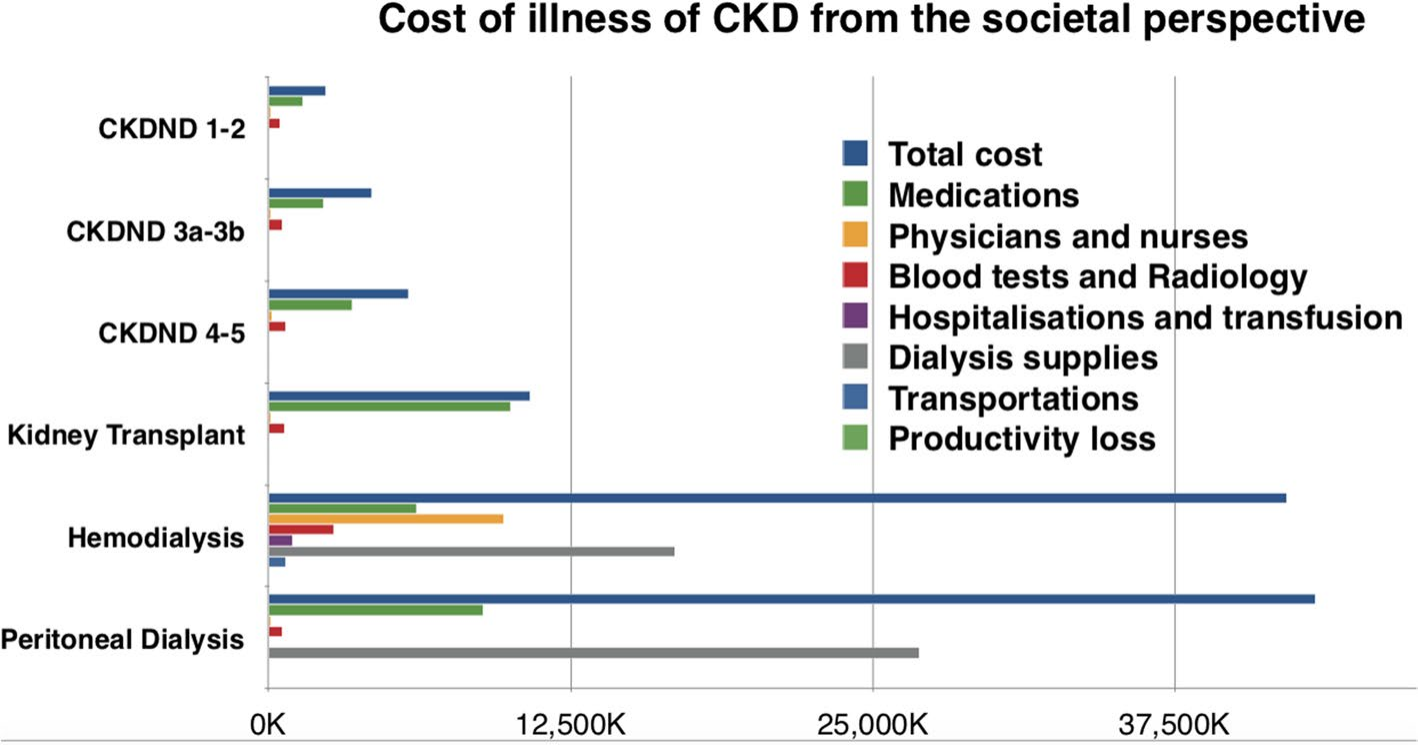
Different components of cost within each category of CKD. Note. Costs are expressed in LBP (1 US Dollar=1515 LBP at the time of this analysis)

From the societal perspective, the median total cost of all categories of chronic kidney disease was estimated at 7,217,500 LBP (3,750,000–35,535,250), with a significant difference between CKD-ND, HD, PD and kidney transplant patients (Table 4). The median opportunity cost or loss of productivity was 0 (0,0) in all categories, CKD-ND, PD and transplant. Four out of 40 HD patients had high opportunity costs and three out of them were < 62 years old. The kidney transplant patients had the highest cost of medications compared to dialysis and CKD-ND patients (Fig. 1 and Figure S1). Hemodialysis patients contributed to the highest cost of physicians, nurses and transportations whereas peritoneal dialysis patients had the highest cost of technique (Fig. 1). When assessing the costs of different stages of CKD-ND, advanced stages of CKD showed to be significantly more expensive with a significant contribution of blood tests and medications to the highest cost (Table 5). The Erythropoietin Stimulating Agents (ESAs) cost was the highest among stage 5 CKD-ND and peritoneal dialysis (Figure S2).

**Table 4:** Costs per year across different kidney disease categories from the societal perspective

COSTS per year	Total *N* = 160	CKD-ND *N* = 102	Transplant *N* = 10	PD *N* = 8	HD *N* = 40	*p-*value*
**Cost of medications**						
Median (IQR)	3,991,000 (2,101,250–7,669,500)	2,688,000 (1,785,000–4,194,750)	10,025,500 (7,086,000–14,400,500)	8,871,000 (7,307,500–10,791,000)	6,150,000 (4,051,500–8,737,500)	< 0.001
**Cost of ESA**						
Median (IQR)	0 (0–1,344,000)	0 (0–0)	0 (0–0)	3,312,000 (1,824,000–3,840,000)	540,000 (0–1,512,000)	< 0.001
**Cost of blood tests**						
Median (IQR)	715,000 (460,000–2,449,000)	600,000 (417,500–800,000)	680,000 (470,000–830,000)	564,000 (451,250–760,000)	2,584,500 (2,487,000–2,800,000)	< 0.001
**Cost of radiology**						
Median (IQR)	0 (0–68,000)	0 (0–75,000)	67,500 (0–127,500)	0 (0–0)	60,000 (60,000–60,000)	< 0.001
**Cost of hospitalizations**						
Median (IQR)	0 (0–1,791,750)	0 (0–857,750)	0 (0–0)	0 (0–1,125,000)	992,500 (0–8,791,750)	0.023
**Cost of transfusions**						
Median (IQR)	0 (0–0)	NA	NA	NA	540,000 (354,000-)	
**Cost of physicians**						
Median (IQR)	165,000 (76,250–4,537,500)	100,000 (50,000–200,000)	90,000 (0–195,000)	100,000 (12,500–160,000)	5,850,000 (5,850,000–5,850,000)	< 0.001
**Cost of nurses**						
Median (IQR)	3,900,000 (3,900,000–3,900,000)	NA	NA	NA	3,900,000 (3,900,000–3,900,000)	
**Cost of transportation**						
Median (IQR)	60,000 (40,000–390,000)	40,000 (20,000–60,000)	55,000 (20,000–100,000)	80,000 (52,500–137,500)	720,000 (720,000–720,000)	< 0.001
**Dialysis modality cost**						
Median (IQR)	0 (0–16,812,000)	NA	NA	26,928,000 (22,896,000–40,680,000)	16,812,000 (16,812,000–16,812,000)	
**Total cost**						
Median (IQR)	7,217,500 (3,750,000–35,535,250)	4,527,500 (2,854,500–6,345,250)	10,825,500 (7,723,000–16,977,250)	43,322,488 (34,203,000–49,648,750)	42,144,500 (36,684,500–53,236,000)	< 0.001

^*^Kruskal–Wallis test was used to compare the different categories of CKD

*NA* Not applicable

**Table 5:** The difference in costs per year between CKD stages from the societal perspective

	**CKD stages 1–2** ***N = 15***	**CKD stages 3a-3b ** ***N* = 42**	**CKD stages 4–5** ***N* = 45**	*p-*value*
**Cost of medications**				
Median (IQR)	1,440,000 (756,000–2,016,000)	2,292,000 (1,797,000–3,657,500)	3,500,000 (2,115,000–5,749,500)	< 0.001
**Cost of ESA**				
Median (IQR)	0	0 (0–0)	0 (0–1,440,000)	< 0.001
**Cost of blood tests**				
Median (IQR)	450,000 (230,000–650,000)	535,000 (345,000–734,750)	690,000 (505,000–900,000)	0.004
**Cost of radiology**				
Median (IQR)	0 (0–137,000)	0 (0–62,500)	0 (0–85,000)	0.759
**Cost of hospitalizations**				
Median (IQR)	0 (0–800,000)	0 (0–134,000)	0 (0–1,800,000)	0.347
**Cost of physicians**				
Median (IQR)	100,000 (50,000–170,000)	100,000 (50,000–200,000)	160,000 (50,000–245,000)	0.126
**Cost of transportation**				
Median (IQR)	20,000 (20,000–40,000)	40,000 (20,000–60,000)	50,000 (40,000–100,000)	0.002
**Total cost**				
Median (IQR)	2,382,000 (1,320,000–4,843,000)	4,281,500 (2,600,250–4,991,000)	5,794,520 (3,556,500–8,628,000)	0.019

^*^Kruskal–Wallis test was used to compare the different categories of CKD-ND

### Costs from the third-party payer perspective

From the third-party payer perspective, the median of the total annual cost was estimated at 5,685,500 LBP (2,281,750–32,386,500) with the peritoneal dialysis patients' costs contributing to the highest expenditure due mainly to the cost of technique and medications (Table 6). Hemodialysis cost included a significantly high proportion of blood tests.

**Table 6:** Costs per year across different kidney disease categories from the third-party payer perspective

COSTS per year	Total *N* = 160	CKD-ND *N* = 102	Transplant *N* = 10	PD *N* = 8	HD *N* = 40	*p-*value*
**Cost of medications**						
Median (IQR)	2,758,500 (909,500–5,590,250)	1,693,500 (0–3,094,500)	7,550,000 (6,046,250–12,223,750)	6,834,000 (3,337,500–8,906,250)	5,530,500 (3,095,250–7,180,500)	< 0.001
**Cost of blood tests**						
Median (IQR)	648,000 (409,500–1,560,000)	510,000 (322,500–748,750)	577,500 (382,500–705,500)	432,500 (103,000–698,500)	1,561,000 (1,500,000–1,748,250)	< 0.001
**Cost of radiology**						
Median (IQR)	0 (0–59,750)	0 (0–66,000)	61,500 (0–108,250)	0 (0–0)	60,000 (60,000–60,000)	< 0.001
**Cost of hospitalizations**						
Median (IQR)	0 (0–1,577,000)	0 (0–834,000)	0 (0–0)	0 (0–1,012,500)	976,500 (0–7,553,500)	0.012
**Cost of physicians**						
Median (IQR)	55,000 (55,000–4,487,500)	0 (0–81,250)	30,000 (0–75,000)	0 (0–56,250)	5,850,000 (5,850,000–5,850,000)	< 0.001
**Cost of nurses**						
Median (IQR)	0 (0–2,925,000)	NA	NA	NA	3,900,000 (3,900,000–3,900,000)	< 0.001
**Dialysis modality cost**						
Median (IQR)	0 (0–16,812,000)	NA	NA	26,928,000 (22,896,000–40,680,000)	16,812,000 (16,812,000–16,812,000)	< 0.001
**Total cost**						
Median (IQR)	5,685,500 (2,281,750–32,386,500)	2,993,500 (1,129,500–5,326,500)	9,173,500 (6,436,000–14,350,250)	39,440,000 (31,705,000–45,635,000)	35,420,500 (32,642,500–42,951,250)	< 0.001

^*^Kruskal–Wallis test was used to compare the different categories of CKD

*NA* Not applicable

### Factors associated with higher costs

When analyzing the sub-category of hemodialysis patients, age was not found to correlate with cost (Spearman's rho correlation coefficient = -0.001, *p* = 0.995) but dialysis vintage negatively correlated with total societal cost (Spearman's rho correlation coefficient = -0.391, *p* = 0.013).

The logistic regression analysis found diabetes as a risk factor for a total hemodialysis cost above the median of 42 million LBP (OR = 2.3; 95%CI: 0.638,8.538; *p* = 0.201). The total societal cost was not significantly different between patients dialyzed with a catheter or fistula (*p* = 0.495, Mann Whitney U test).

In the subcategory of CKD-ND patients, age correlated with the total societal cost (Spearman's rho correlation coefficient = 0.323, *p* = 0.001). Diabetes and coronary artery disease were significantly associated with a total CKD-ND cost above the median of 4.5 million LBP (OR = 2.4; 95%CI: 1.083,5.396; *p* = 0.031; OR = 3.7; 95%CI: 1.535,8.938; *p* = 0.004 respectively). The median total societal cost of patients with urine albumin to creatinine ratio < 300 mg/g was 4,120,000 LBP (2,447,500–5,849,260) and of those > 300 mg/g 5,024,000 LBP (3,500,825–8,480,500) (*p* = 0.045, Mann Whitney U test).

## Discussion

This study shed lights on the high cost of dialysis compared to non-dialysis chronic kidney disease and kidney transplant. Dialysis is known to be a life-saving renal replacement therapy but it comes with a very high financial burden [13]. Health systems worldwide carry variable capacities when dealing with the high cost of dialysis [20]. Both hemodialysis and peritoneal dialysis are modalities that necessitate chronic use of expensive medical supplies and high-cost healthcare services. However, when comparing the annual dialysis treatment cost among our sample of patients, the cost of PD bags outweighed the HD treatment supplies including filters, bicarbonate cartridge, tubing, fistulas, acid dialysate and pyrogen filters; on the other hand, HD compared to PD entailed higher hospitalization rates, higher cost of transportation and higher healthcare providers' cost such as physicians and nurses. The Lebanese MOPH reimburses the physicians' fees for HD but not for PD. This practice leads to less follow-up of PD by physicians and less cost. It is difficult to compare the cost of dialysis in Lebanon to other reported costs globally. A systematic review of dialysis costs in low and middle-income countries has shown a great variability in costs and in methodology when assessing costs [21]. Most of the studies were not clear about the perspective used and very few included direct and indirect costs. This meta-analysis highlighted as well that dialysis is mostly cost-effective in upper middle-income countries but not in low resources countries [21]. In addition, the majority of studies reported similar expenditures whether patients were on hemodialysis or peritoneal dialysis. In fact, a multinational study published in 2013 showed that PD costs can be reduced in developed countries but it was rather complicated in LMIC countries [22]. On the other hand, hemodialysis costs can be reduced by decreasing the number of sessions per week in low resources settings [23, 24]. In our sample, all hemodialysis patients were treated with thrice-weekly dialysis. A scenario with twice-weekly dialysis for patients with residual kidney function can help lower the total cost of hemodialysis and lighten the cost burden on society and third-party payers. It is very peculiar that our study showed less cost with longer dialysis vintage. The only plausible explanation is that these patients who lived longer on dialysis had less comorbidities, less hospitalizations and they had the survival advantage of healthier patients.

Interestingly, our study has shown that kidney transplantation is four times less expensive than dialysis when taking into consideration median annual costs. However, transplantation is associated with a high burden of medications' cost. Several strategies have been used worldwide for cost-saving especially regarding medication induction [25]. Efforts are needed to release affordable and high-quality immunosuppressive drugs in collaboration with pharmaceutical companies that have been leaders in the production of these medications.

CKD is the least expensive at the first stages. The cost of medications increases in advanced stages for several reasons. First, the number of medications is significantly higher in CKD stages 4 and 5 as shown by our study, second these patients need ESA for their anemia which is expensive and third they need more antihypertensive drugs and doses of diuretics to manage the hypertension and sodium retention. This increase of cost across CKD stages calls for interventions that could slow kidney disease progression. Decreasing unnecessary hospitalizations can lower the cost of CKD [26]. A good follow-up of diabetic patients can prevent the progression of diabetic nephropathy [27], knowing that diabetes is causing 10% of the global health expenditure and kidney disease is one of the most expensive diabetic complications [28]. Our study has highlighted the impact of some factors such as age, diabetes, albuminuria and coronary artery disease on the total cost in CKD-ND patients. Health policies should target preventive measures of coronary artery disease that can ultimately reduce the societal cost of CKD. Another strategy to reduce the progression of CKD and its related cost is the timely referral of CKD patients to nephrologists.

The loss of productivity as part of the opportunity cost was not very high in our sample. First, transplantation prevents young patients from losing their jobs and thus prevents the productivity loss. The hemodialysis patients are in the majority elderly and most of them are retired. The CKD-ND patients are young and productive at the first stages of CKD, and they are retired at advanced stages when the disease becomes a burden.

Finally, our report has revealed a great percentage of CKD-ND patients paying out-of-pocket for their disease treatment and follow-up. The out-of-pocket for medications is reduced in the advanced stages of kidney replacement therapy such as dialysis and kidney transplant but it is still contributing to blood tests, transportations, a part of hospitalizations and thus it is still a high burden on the patient and the family. It is reflected in the difference between the total costs from the societal and third-party payer perspectives.

Our study has some limitations. It is retrospective and it is based on a sample of the population because Lebanon has no registries or database for chronic kidney disease patients. It also included small numbers of PD and kidney transplant patients. Despite the small number, the PD patients had a wide range of age and dialysis vintage, and the price of bags for a standard peritoneal dialysis is the same for all patients in the country. We believe that the cost estimated for PD patients reflects well the reality. Similarly for transplant patients, their years of transplant ranged between one and 21 years. Transplant patients within the first year have higher costs because of higher doses of immunosuppression and possible higher rates of hospitalizations. We believe as well that the cost estimated from our study gives an estimation very close to reality.

Our study's main strength is that it is the first one in Lebanon and in the region to assess all components of cost among different categories of chronic kidney disease patients.

In conclusion, this cost of illness study showed a high burden of hemodialysis and peritoneal dialysis cost compared to transplant and non-dialysis CKD patients. It also revealed a high cost of medications in transplant patients. Health policies should target interventions that prevent end-stage kidney disease and encourage kidney transplantation.

## Supplementary Information

**Additional file 1: Figure S1**. The total cost of medications per year across different categories of CKD. **Figure S2**. The total cost of ESAs per year across different stages of CKD.

## Data Availability

All data generated or analysed during this study are included in this published article.

## Abbreviations

CKD: Chronic Kidney Disease
LBP: Lebanese Pounds
eGFR: Estimated Glomerular Filtration Rate
CKD-ND: Chronic kidney disease non-dialysis
PD: Peritoneal dialysis
HD: Hemodialysis
MOPH: Ministry of public health
OOP: Out-of-pocket
NSSF: National social security fund
PKD: Polycystic kidney disease
CAD: Coronary artery disease
ESA: Erythropoietin-stimulating agent
