# Correction: Cost of illness of chronic kidney disease in Lebanon: from the societal and third-party payer perspectives

**DOI:** 10.1186/s12913-022-08159-z

**Published:** 2022-06-10

**Authors:** Mabel Aoun, Elie Helou, Ghassan Sleilaty, Rony M. Zeenny, Dania Chelala

**Affiliations:** 1 Department of Nephrology, Faculty of Medicine, Saint-Joseph University, Beirut, Lebanon; 2 Department of Nephrology, Saint-George Hospital, Ajaltoun, Lebanon; 3 Department of Urology, Faculty of Medicine, Saint-Joseph University, Beirut, Lebanon; 4 Unit of biostatistics, Faculty of Medicine, Saint-Joseph University, Beirut, Lebanon; 5 Pharmacy Director, American University of Beirut Medical Center, Beirut, Lebanon; 6 Department of Nephrology at Hotel-Dieu de France Hospital, Faculty of Medicine, Saint-Joseph University, Beirut, Lebanon

**Correction: BMC Health Serv Res 22, 586 (2022)**

**https://doi.org/10.1186/s12913-022-07936-0**

Following publication of the original article [1], the authors identified an error in the first paragraph of the ‘General characteristics’ subsection under the ‘Results’.

The sentence originally read:

The median dialysis vintage of PD and HD patients were 36 **years** (29,49) and 49 **years** (26.5,96) respectively and they ranged between 13 and 50 months for PD and one and 21 years for HD.

The sentence should read:

The median dialysis vintage of PD and HD patients were 36 **months** (29,49) and 49 **months** (26.5,96) respectively and they ranged between 13 and 50 months for PD and one and 21 years for HD.

The original article [1] has been corrected.
