# Supplementary material for: Cost of illness of chronic kidney disease in Lebanon: from the societal and third-party payer perspectives
Source: BMC Health Serv Res. 2022 May 1;22:586. doi: 10.1186/s12913-022-07936-0 (PMC9063193; doi:10.1186/s12913-022-07936-0)
Supplement: Supplementary file 1 — Additional file 1: Figure S1. The total cost of medications per year across different categories of CKD. Figure S2. The total cost of ESAs per year across different stages of CKD. [file 12913_2022_7936_MOESM1_ESM.docx]

**Figure S1. The total cost of medications per year across different categories of CKD**


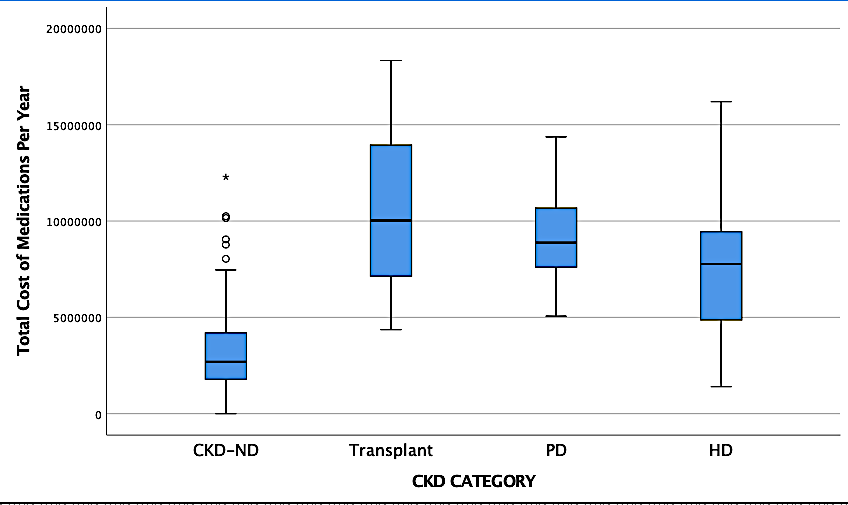


Note. CKD-ND, chronic kidney disease non-dialysis; PD, peritoneal dialysis; HD, hemodialysis.

Costs are expressed in LBP (1 US Dollar=1515 LBP at the time of this analysis).

**Figure S2. The total cost of ESAs per year across different stages of CKD**

**
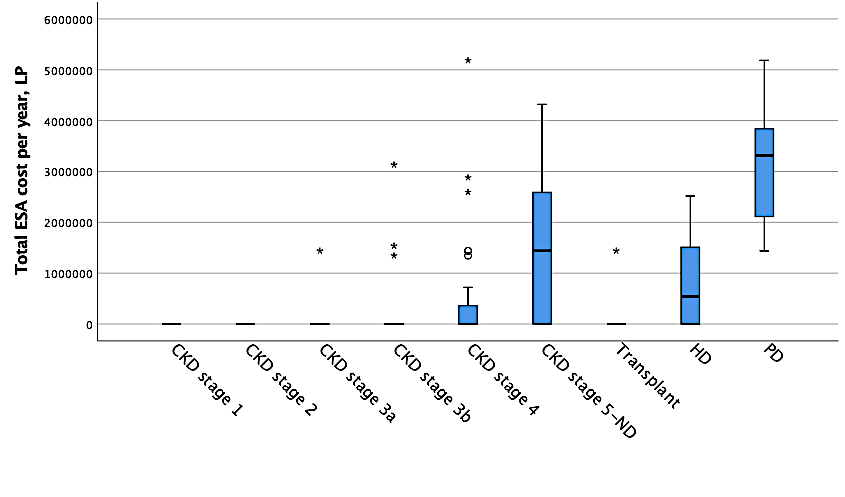
**Note. CKD-ND, chronic kidney disease non-dialysis; PD, peritoneal dialysis; HD, hemodialysis. Costs are expressed in LBP (1 US Dollar=1515 LBP at the time of this analysis).
